# Life-threatening Anaemia in Patient with Hereditary Haemorrhagic Telangiectasia (Rendu-Osler-Weber Syndrome)

**DOI:** 10.1515/med-2020-0020

**Published:** 2020-03-06

**Authors:** Melania Mikołajczyk-Solińska, Karolina Leończyk, Aleksandra Brzezina, Sylwia Rossa, Jacek Kasznicki

**Affiliations:** 1 Department of Internal Medicine, Diabetology and Clinical Pharmacology, Medical University of Lodz, 251 Pomorska Street, 92-213 Lodz, Poland; 2 Central Teaching Hospital of the Medical University of Lodz, 251 Pomorska Street, 92-213 Lodz, Poland

**Keywords:** Rendu-Osler-Weber syndrome, telangiectasias, epistaxis, anaemia, haemostasis

## Abstract

Hereditary haemorrhagic telangiectasia (HHT), also known as Rendu-Osler-Weber syndrome, is a rare autosomal dominant vascular disorder. Patients with HHT may present with a wide spectrum of clinical manifestations from epistaxis to clinically significant arteriovenous malformations (AVM) in the lungs, liver, brain and spine. The diagnosis of HHT is based on clinical criteria. There is a long diagnostic delay of nearly 3 decades since disease onset. The treatment is based on various types of haemostasis. There is ongoing research with potential therapies which may prevent and decrease the severity of epistaxis. Thalidomide may be an effective treatment to decrease the bleeding symptoms of patients with HHT.

## 1 Background

Hereditary haemorrhagic telangiectasia (HHT) or Rendu-Osler-Weber syndrome, is a disorder characterised by mucocutaneous telangiectasias and arteriovenous malformations (AVM) of internal organs. It is a rare autosomal dominant disorder that occurs in 1/5000-1/8000 individuals [4]. A clinical spectrum of HHT varies from asymptomatic and incidentally detected lesions, aesthetic problems due to facial telangiectasias to episodes of recurrent epistaxis, gastrointestinal or urinary tract bleeding. On certain occasions, due to AVM, a wide range of serious complications may occur including pulmonary arterial hypertension, high output heart failure, liver failure, portal hypertension, cerebral abscess and stroke. Patients with HHT present with various manifestations of the disease and the symptoms intensify gradually with age [12].

A diagnosis of HHT is based on clinical Curacao criteria: 1. Spontaneous recurrent epistaxis, 2. Multiple telangiectasias in typical locations, 3. Proven visceral AVM (lungs, liver, brain, spine), 4. First-degree family member with HHT. If three or four criteria are met, a patient has “definite HHT”, two gives “possible HHT”, if only one criterion is present a diagnosis is unlikely [13].

There are 5 genes associated with HHT. The dominant genes are: endoglin (ENG) and activin-like receptor kinase 1 (ALK1/ ACVRL1) genes are the major ones. Mutations in a third gene known as MADH4 may cause HHT and juvenile polyposis. The function of the two other genes are still unknown. The three known genes are elements of the transforming growth factor-beta (TGF-b) signalling pathway, which regulates cell differentiation and proliferation. The TGF-b signalling pathway plays a key role in pathogenesis of HHT and leads to vascular dysplasia and malformation. McDonald et al. demonstrated that 96% of patients who fulfilled 3 or 4 Curacao criteria had an ENG or ALK1 mutation [8].

## 2 Case presentation

A 55-years old male patient, pensioner was admitted to the Department of Internal Medicine, Diabetology and Clinical Pharmacology of Medical University of Lodz, Poland, due to progressive, generalized weakness, pale skin and frequent nosebleeds. The patient denied “coffee ground” vomiting, tar coloured stool and other bleedings. Medical history revealed appearing recurrent epistaxis since age 40.

**Figure 1:**
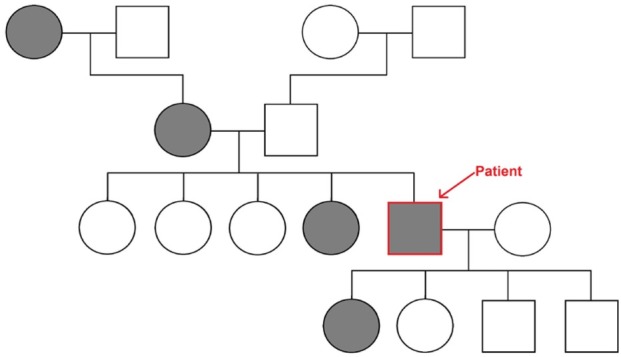
Pedigree of the family

In past medical history we documented bronchial asthma, condition after removal of the ascending aorta aneurysm and replacement of the aortic valve (2011), condition after ischemic stroke (2013, 2014), condition after operation of both side inguinal hernias (2006, 2016). Patient took following medications: budesonide + formoterol 320 ug + 9 ug – 1 inhalation twice a day, acenocumarol 4 mg – 0.5 tablet once a day, bisoprolol 5 mg - 0.5 tablet once a day, tranexamic acid 500 mg – 1 tablet three times a day. The patient denied allergies and stimulants. In his family history, the patient’s grandmother, mother, sister and one daughter presented with frequent, recurrent epistaxis. Pedigree of the family was presented on Figure 1.

**Figure 2:**
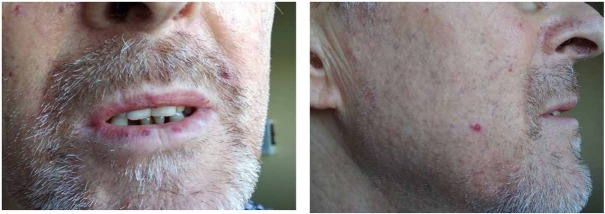
A, B. Facial telangiectasias.

On admission the patient was alert and cooperative. Physical examination revealed pale skin, and haemorrhagic telangiectasias on the lips and cheeks (Figure 2 A, B). Temperature was 36.6^⍛^C, pulse rate was 100 per minute, arterial blood pressure was 100/60 mmHg. The breath sounds were normal. On cardiac examination audible click of the artificial aortic valve was present. The abdomen was soft, painless, liver and spleen were impalpable, peristalsis was audible, peritoneal symptoms were absent. The Goldflam sign was negative on both sides. Oedema was absent. The patient did not agree to a digital rectal examination.

ECG indicated steady sinus rhythm, 100 beats per minute, normal axis, flat-negative T wave in lead III.

Basic laboratory tests revealed significant deviations: red blood cells (RBC) 3.09*10^6/uL (reference values 4.20-6.10), haemoglobin, HGB 5.5 g/dl (14.0-18.0), haematocrit (HCT) 20.5 % (40.0-55.0), mean corpuscular volume (MCV) 66 fl (80-98), mean corpuscular haemoglobin (MCH)17.8 pg (26.0-34.0), Iron 1.6 umol/l (11-33), Ferritin 5 ng/ml (22-322), total iron binding capacity (TIBC) 72.6 umol/l (45-70), international normalized ratio (INR) 1.78 (0.80-1.20). Glomerular filtration rate (GFR) calculated with Chronic Kidney Disease Epidemiology Collaboration (CKD-EPI) equation was 73.0 ml/min/1.73m^2^. Other parameters were within the normal ranges.

Presented laboratory parameters were measured by following methods: RBC - impedance and flow cytometry, HGB - photometric method using sodium lauryl sulfate, HCT - cumulative counting of electrical pulses, MCV and MCH - calculated data resulting from direct measurements, Iron - colorimetric analysis with 2,4,6-Tris(2-pyridyl)-s-triazine, Ferritin - electro-chemiluminescence immunoassay method, TIBC - colorimetric method with ferrozine. Prothrombin time – needed to calculate INR - was assessed with coagulation method and nephelometric measurement. Serum creatinine - used to calculate GFR (CKD-EPI)-was measured with enzymatic-colorimetric method.

A chest X- ray showed no deviations. An ultrasound examination of abdomen revealed 7 mm cyst in right kidney and 8 mm cyst in left kidney. A gastroscopy demonstrated erosive inflammation of the mucous membrane in the stomach and duodenum. The patient did not agree to a colonoscopy examination. Transthoracic echocardiography revealed condition after replacement of the aortic valve, mild mitral valve insufficiency, mild pulmonary valve insufficiency, mild tricuspid valve insufficiency, ejection fraction 63%.

During hospitalization the patient required blood transfusion and iron supplementation. Four units of red blood cells concentrate, group A Rh negative, were transfused to the patient. An 800 mg of iron was given intravenously. Parameters of red blood cells were improved: RBC 4.01*10^6/uL, HGB 8.6 g/dl, HCT 29.8 %, MCV 74 fl, MCHC 21.4 pg. The patient was discharged in good condition after eight days of hospitalization.

Patients consent: A written informed consent was obtained from the patient for publication of the case report, including the photos.

## 3 Discussion

Iron deficiency anaemia is a frequent complication of HHT secondary to blood loss. Pahl et al. conducted a retrospective chart review of HHT patients. A total of 168 subjects were included, of which 84 had documented anaemia. The most common was mild anaemia (52%), followed by moderate (37%), and severe anaemia (11%). Epistaxis was the most common cause of anaemia in the mild and moderate groups (75% and 59% respectively), while both epistaxis and gastrointestinal bleeding were present in the majority of patients with severe anaemia (44%) [9]. In our patient the episodes of recurrent epistaxis were the cause of life-threatening anaemia. Therefore, the recurrent epistaxis should be dealt seriously in HHT patients.

Moreover, the patient required anticoagulant therapy despite being at the high risk of bleeding. Patients with artificial heart valves are obliged to take vitamin K antagonists (VKAs). Doctors need to inform patients about medicines and food products that interact with VKAs, as well as self-monitoring devices to check INR in the out-patient environment. Large clinical trials have shown that non vitamin K oral anticoagulants (NOAC) have less intracranial bleeding than VKAs [1]. In HHT patients and other conditions requiring anticoagulation such as venous thromboembolism and/or atrial fibrillation heparin and VKAs remain first choice anticoagulants. If NOAC are considered, apixaban is associated with lower bleeding risk than rivaroxaban. Most of the HHT patients can be treated safely with anticoagulants, however patients must be aware, trained and under constant medical care [14].

Despite the fact that according to Curacao criteria only clinical symptoms are needed to establish diagnosis, researchers observed a long diagnostic delay of approximately 3 decades in patients with HHT since onset of the disease. The main reason for diagnostic lag is the rarity of the disease, heterogeneity of clinical symptoms, and the insufficient knowledge of physicians. Moreover, HHT tends to wait silent for decades before appearing as sudden, acute life-threating events. In most studies first presentation of the disease is isolated epistaxis. Patients usually regarded the nosebleed as a casual ailment for many years rather than perceiving it as the symptom of an underlying disease [10].

Permanent, effective cure for HHT is not available. The treatment is based on symptom relieving. Physicians usually treat the bleeding with various types of haemostatic techniques, such as emergency techniques of locally applied pressure, nasal packing anteriorly and/or posteriorly, electrical cauterization, photocoagulation astringent, intravascular embolization, and surgery. The efficacy of these methods is limited, and has a high rate of recurrence [11]. Another medical treatment is pharmacotherapy with the haemostatic agent tranexamic acid. Patients taking tranexamic acid at a dose of 3 g/day had decrease durations of average daily bleeding compared to patients taking placebo. However, tranexamic acid had no effect on patient haemoglobin level and many patients in the tranexamic acid treatment group reported common side effects such as vertigo and diarrhoea [3]. Research into the pathophysiology of HHT has led to the development of potential therapies that prevent and decrease the severity of bleeding. Several drugs have been investigated in HHT, such as tamoxifen (antiestrogen medication) [15], bevacizumab (an antivascular endothelial growth factor monoclonal antibody) [7], and thalidomide (an antiangiogenic and immunomodulatory agent) [6]. Thalidomide seems to be the best promising treatment in HHT. It may modulate the activation of mural cells and enhance both their proliferation and ability to embrace blood vessels, which means that thalidomide can make HHT vessels more firm and less prone to breaking. A prospective phase II clinical trial analysed the efficacy of thalidomide for severe recurrent epistaxis in HHT and documented that thalidomide could effectively reduce epistaxis, frequency of blood transfusion and improve general condition [6]. Another prospective study documented that thalidomide was effective in improving epistaxis severity and anaemia, but did not reduce gastrointestinal and hepatic bleeding. On the other hand, side effects of thalidomide such as constipation, neuropathy, and thromboembolism may sometimes limit its use in clinical practice [5].

The prognosis for HHT varies and depends on the severity of symptoms. Life expectancy in patients with HHT is lower than in the general population. Sepsis and cardiac failure are the main causes of death [2]. Continuous medical education and close collaboration among different specialities are crucial for early diagnosis and appropriate management.
